# Pothead or pot smoker? a taxometric investigation of cannabis dependence

**DOI:** 10.1186/1747-597X-1-22

**Published:** 2006-08-10

**Authors:** Thomas F Denson, Mitch Earleywine

**Affiliations:** 1 Department of Psychology, University of Southern California, SGM 501, Los Angeles, CA, 90089-1061, USA; 2 Department of Psychology, University at Albany, State University of New York, Social Sciences 369, 1400 Washington Ave., Albany, NY 12222, USA

## Abstract

**Background:**

Taxometric methods were used to discern the latent structure of cannabis dependence. Such methods help determine if a construct is categorical or dimensional. Taxometric analyses (MAXEIG and MAMBAC) were conducted on data from 1,474 cannabis-using respondents to the 2001–2002 National Epidemiologic Survey on Alcohol and Related Conditions (NESARC). Respondents answered questions assessing *DSM-IV *criteria for cannabis dependence.

**Results:**

Both taxometric methods provided support for a dimensional structure of cannabis dependence.

**Conclusion:**

Although the MAMBAC results were not entirely unequivocal, the majority of evidence favored a dimensional structure of cannabis dependence.

## Background

No publication has been more influential in the study and treatment of psychopathology than the *Diagnostic and Statistical Manual of Mental Disorders *(4^th ^ed.; *DSM-IV*) [1]. Criteria for over 300 mental disorders appear in its pages. The *DSM-IV *implies that mental disorders form discrete categories. Many authors have criticized this assumption [2,3]. For example, Widiger and Clark discussed the difficulty in determining threshold criteria for the various disorders and the lack of sufficient empirical support for the existence of the categories [3]. Briefly stated, the "dimensional vs. categorical" debate addresses whether psychopathology is a matter of qualitative differences or a matter of degree of pathology. Proponents of the dimensional view state that mental disorders essentially vary in degree with adaptive functioning, perhaps along multiple dimensions. In contrast, proponents of the categorical view (as represented in the *DSM*) present mental disorders as qualitatively distinct categories within which there is a certain amount of variation by degree. For example, in the current research context, a categorical view would posit that cannabis users who meet dependence criteria are qualitatively different from users who do not become dependent (although there is some variability within the two groups). A dimensional view would posit a continuous degree of cannabis dependence with users falling at various points along this continuum.

Recent advances in statistical methods have provided a powerful tool for determining whether psychological constructs are categorical or dimensional (i.e., determining the *latent structure*). Meehl and colleagues have developed a number of these statistical methods, known as *taxometrics *[4,5]. Taxometric procedures allow one to determine whether constructs are best conceived of as dimensional or whether they are a natural "type" (i.e., category). These procedures are different from traditional methods such as factor analysis or cluster analysis because taxometrics do not impose a structure on the data. Taxometric procedures have been applied to many psychological constructs (for a review, see [6]) such as depression [7-9], anxiety [10], psychopathic personality [11,12], posttraumatic stress disorder [13], schizotypy [14], worry [15], sexual orientation[16], and marital discord [17].

To our knowledge, however, no study has investigated the latent structure of any of the substance dependence disorders. Thus, the current study sought to obtain empirical evidence as to whether there are qualitative differences between cannabis users who are dependent on the substance and those who are not, or whether cannabis dependence is better conceptualized as a dimension. Although the *DSM *suggests that the latent structure of cannabis dependence is categorical, this has not been empirically examined.

Why is the latent structure of cannabis dependence important? This distinction has important implications for understanding policy, treatment, and assessment of cannabis dependence. Regarding policy, if cannabis dependence truly forms a latent category, prevention and treatment resources should be directed primarily at those users who already meet criteria for dependence or are most likely to develop dependence. On the other hand, if cannabis dependence is dimensional, these same resources should target all users or users with a single symptom.

Two recent cross-sectional population-based surveys of cannabis users found rates of dependence for young users at 7% and adult users at 21% [18,19]. Given the relatively high prevalence of the disorder among cannabis users, determining the latent structure of cannabis dependence (and other substance dependences) may be an important first step in providing improved treatment. For example, rather than waiting until a client meets full *DSM *criteria to initiate treatment, a dimensional view would suggest that treatment could be most helpful if initiated as soon as one develops one or two dependence symptoms and before the onset of additional symptoms.

The latent structure of cannabis dependence also has implications for assessment in research and clinical settings. If the distinction between dependent and non-dependent is a true category, then two-group comparisons between these categories might prove the most powerful and appropriate. Any variation within the dependent and non-dependent groups would likely be less relevant. In contrast, a dimensional model would suggest that continuous variables like symptom counts or severity of problems might be a more appropriate approach.

### The current research

In the current study, we present evidence for the dimensional latent structure of cannabis dependence. The current research offers a number of strengths. First, our sample was obtained from the National Epidemiologic Survey on Alcohol and Related Conditions (NESARC). This fairly large sample is representative of the United States population. Cannabis users in our sample varied from everyday users to once in the last twelve months. By including a wide variety of cannabis users we enhanced our ability to detect a latent categorical structure, if such a structure truly exists (see [5]). Thus, all cannabis users were included in the sample whether dependent or not. Second, the items used in the NESARC are based directly on *DSM-IV *criteria. By including such items as our indicators, we can be fairly confident that our conceptualization of cannabis dependence is highly similar, if not identical, to the conceptualization of cannabis dependence in the *DSM-IV*. Third, we included simulated comparison data sets in our analyses. Simulated data provide improved accuracy by taking into account the skew of the actual data. In the absence of simulated data, skewed data may lead one to erroneously infer the existence of a low frequency 'condition' believed to be a disease category (i.e., taxon).

## Results and discussion

We analyzed data from the 1,474 participants with complete data (31 missing cases). Participants who endorsed three or more of the seven symptoms of cannabis dependence (thus meeting *DSM-IV *criteria) were included in the taxon group. Those who endorsed zero to two symptoms were considered members of the complement group. Using this criterion resulted in a taxon group of 291 individuals and a complement group of 1,183. Thus, 20% of the sample was in the putative taxon group, twice as large as the 10% deemed sufficient in Monte Carlo studies to detect a categorical distinction, should one exist [4,20,21].

We first examined the mean correlations among the seven indicators in the taxon and complement groups (e.g., "nuisance" correlations; see [5]). Ideally, these correlations should be close to zero and this was indeed the case for the taxon, mean *r *= .03, *SD = *.11, and complement groups, *r *= .03, *SD *= .05. The average correlation in the total sample, where larger correlations are desired, was adequate, mean *r *= .25, *SD *= .06 [5]. Although total sample correlations are not routinely reported in the taxometric literature, a survey of recent articles revealed that our total correlation was within the small to moderate range reported in recent articles. For instance, Strong et al. reported a whole-sample correlation of .32, whereas Edens et al. reported a whole sample correlation of .36 [9,11]. Moreover, we used dichotomous measures, which are often associated with less variability than ordinal measures. In addition, our nuisance correlations are quite a bit smaller than those typically reported in the literature, thus enhancing our ability to detect a categorical distinction, should one exist. The standardized validities for the individual items were 2.10, 2.04, 2.79, 0.89, 1.47, 2.12, and 2.04. The mean indicator validity in standard deviation units was 1.92. This value was above the 1.25 mean indicator validity recommended as acceptable [22].

Because taxometric methods do not rely on standard hypothesis testing, we used three criteria to judge the latent structure of cannabis dependence. First, we visually inspected the individual and averaged taxometric curves and compared them with the averaged curves for the simulated dimensional and taxonic data sets. Second, we evaluated the base rate estimates for each of the procedures. Taxonic results generally produce a narrow range of base rate estimates, whereas dimensional results generally produce a wide range of base rate estimates. Third, we examined a goodness-of-fit index to examine whether the research data more closely resemble the simulated taxonic or dimensional data. This statistic is the root mean square residual (*Fit*_*RMSR*_). Smaller values indicate better fit.

### MAXEIG analyses

**Figure 1:**
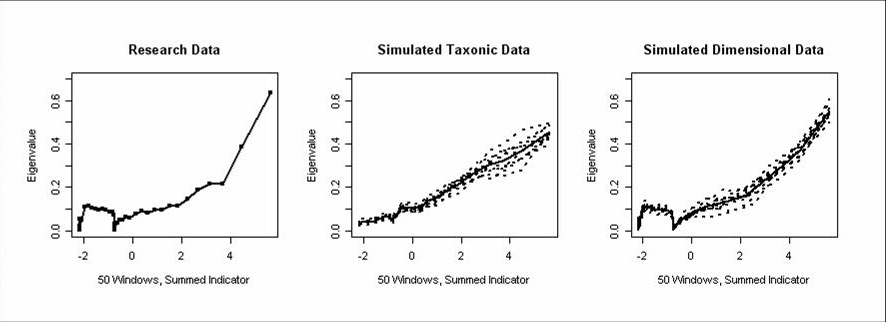
The averaged MAXEIG curves for the research data, simulated taxonic data, and simulated dimensional data. The simulated data was based on 10 simulations. The x-axis represents a composite of 5 of the 7 cannabis dependence symptoms ordered and divided into 50 sections (i.e., windows) with 90% overlap. This is the input variable. The covariance between the remaining two indicators (output variables) is plotted on the y-axis for each window. This process was repeated for all possible input-output combinations. All variables were standardized.

None of the 21 MAXEIG curves revealed a definitive inverted U shape, suggesting that the latent structure of cannabis dependence may be dimensional. The average curve failed to reveal an inverted U shape and actually revealed a somewhat concave shape. The average MAXEIG curve and the average curve for the dimensional and taxonic simulated data sets are presented in Figure 1. Base rates varied widely from .015 to .940 (*M *= .09, *SD *= .20), also suggesting a dimensional structure. Furthermore, the *Fit*_*RMSR *_value for the 10 simulated taxonic data was 3.13 times as large as the 10 simulated dimensional data sets, .058 and .019, respectively. Taken together, these results offer support for a dimensional structure of cannabis dependence.

### MAMBAC analyses

**Figure 2:**
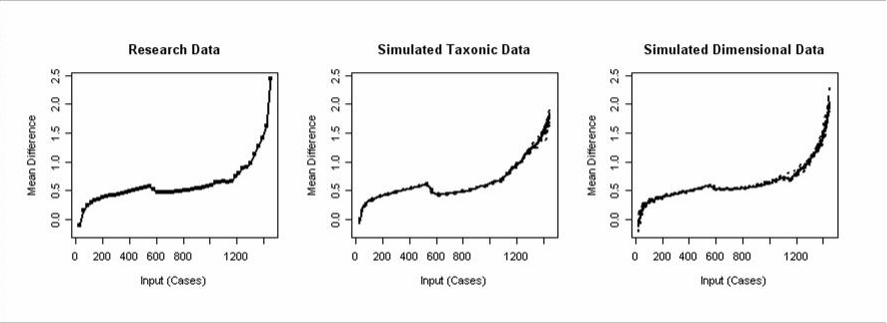
The averaged MAMBAC curves for the research data, simulated taxonic data, and simulated dimensional data. The simulated data was based on 10 simulations. The x-axis represents the average of all possible composites of 6 of the 7 indicators ordered and cut at 50 intervals. This is the input variable. The variable not included as part of the input variable composite served as the output variable. At each cut, mean scores for those cases above and below the cut value were calculated. This mean difference is plotted on the y-axis. All variables were standardized.

Only one of the curves had a taxonic appearance. The remaining curves were concave in appearance. The average curve also had a concave appearance, consistent with a dimensional latent structure. The average MAMBAC curve and the average curve for the 10 dimensional and 10 taxonic simulated data sets are presented in Figure 2. Although the average curve appears slightly more similar to the simulated dimensional data than the simulated taxonic data, the degree of similarity is not overwhelmingly in favor of a dimensional conceptualization, perhaps due to the limited variability associated with dichotomous measures. Thus, these data may not meet strict suitability test criteria. Nonetheless, the remaining evidence was consistent with a dimensional interpretation. As was the case with the MAXEIG analyses, base rate estimates varied considerably from .000 to .524 (*M *= .12, *SD *= .19), suggesting a dimensional structure. Also in support of a dimensional structure, the *Fit*_*RMSR *_value for the taxonic data sets was 1.53 times as large as the value for the dimensional data sets, .12 and .08, respectively. In conclusion, these data provide further support for a dimensional latent structure of cannabis dependence.

## Conclusion

This was the first study to our knowledge to use taxometric methods to assess the latent structure of a substance dependence disorder. Contrary to the prevailing trend to consider those who meet *DSM *criteria for cannabis dependence as qualitatively distinct from other cannabis users, the current research provided support for the conceptualization of cannabis dependence as a dimension. Two nonredundant taxometric procedures (MAXEIG and MAMBAC) revealed an identical pattern of results in support of the dimensional conceptualization (although the curves for the MAMBAC procedure were somewhat less unequivocal than the MAXEIG curves). Base rate estimates also varied widely, suggesting a dimensional structure. Moreover, the representativeness of our sample lends confidence to the generalizability of these results.

A dimensional structure has implications for treatment and prevention of cannabis dependence. Dimensional rather than categorical progress in treatment has considerable intuitive appeal. Gradual improvements and incremental decreases in problems might prove more likely than categorical shifts between problem use and complete abstinence. The dimensional model might also alter our approaches to prevention of cannabis dependence. Given the quantitative rather than qualitative differences between problematic and harmless use, strategies that target regular cannabis users who are uninterested in abstinence might have the potential to create considerable benefit. Finally, given this dimensional model, continuous measures of symptom severity might have more statistical power than dichotomous measures that simply assess group membership. Such measures may prove useful in research and treatment settings.

Future studies could address other common substance dependence disorders such as alcohol, opiate, cocaine, and methamphetamine. Improved understanding of the latent structure of substance dependence has important implications for the treatment of substance users. If treatment is initiated at the early stages of symptom appearance, it is likely that substance abusers might be prevented from developing additional and potentially more incapacitating symptoms. For example, public service announcements could persuade cannabis users to seek treatment as soon as they find themselves using more than desired or developing a tolerance. It is likely that the prognosis would be favorable under these circumstances.

## Method

### Participants

Participants were 1,505 individuals (62% male; *M *age = 31.22, *SD *= 11.03 years) who completed the 2001–2002 NESARC survey and reported using cannabis both during the past 12 months and earlier. In order to provide for an adequate proportion of dependent individuals, if participants had only used it in the past 12 months (and not earlier) they were not included. The NESARC is sponsored by the National Institute on Alcohol Abuse and Alcoholism (NIAAA) and contains a representative sample of the United States population. We thus had an adequate sample size (> 300 cases) to conduct taxometric analyses [22]. Participants reported their age of heaviest use at an average age of 20.9 years (*SD *= 11.8).

### Materials

As part of a larger survey on drug and alcohol use, participants completed a series of dichotomous (yes/no) questions assessing *DSM-IV *criteria for cannabis dependence. These questions assessed tolerance, withdrawal symptoms, using more than intended, desire for the drug, spending more time than desired acquiring or using the drug, giving up social or pleasurable activities, and physical or psychological problems associated with cannabis use. These seven criteria served as the seven indicators for the taxometric analyses.

### Procedure

Since replication across more than one taxometric procedure lends confidence to our conclusions, our analytic strategy consisted of two taxometric methods developed by Meehl and colleagues: MAXEIG and MAMBAC [4,5]. These procedures use quantitative methods to determine whether qualitative differences exist in the latent structure (i.e., taxonic or dimensional) of cannabis dependence. We used programs written by J. Ruscio for the R software package [23]. For each method, we created 10 dimensional and 10 taxonic simulated data sets based on the distributional features of the research data. Since skew may produce misleading results (e.g., peaks in the absence of a taxonic latent structure) [see [8,13]], simulated data provides advantages because it enables comparison of the taxometric results between the research data and simulated data with similar skew and kurtosis. Such parallel analyses with simulated data are now standard in the taxometric literature [8,12,13,17].

MAXEIG is a multivariate extension of the maximum covariance method (MAXCOV) [5]. In the present analyses, the input variable refers to the degree of cannabis dependence. Whereas with MAXCOV, one indicator serves as an input variable (x) and the remaining two indicators serve as output variables (y), J. Ruscio's MAXEIG program allows for the simultaneous analysis of multiple variables [23]. Thus, the current data were ordered according to a sum of five of the seven dependence items (i.e., the input variable) into 50 sections overlapping by 90% (i.e., windows with 90% overlap). Two items were removed to serve as output variables. The covariance of the two output variables is then plotted on the y-axis for each of the 50 windows across the x-axis. This process was repeated for all additional combinations of two output variables. Taxonic figures generally have a peak where the taxon and complement groups are most evenly divided. Dimensionality is generally indicated by a concave, irregular, or flat figure. Variables were standardized for the analyses.

We also used the Mean Above Minus Below a Cut (MAMBAC) method as our second nonredundant taxometric procedure [5]. The concept underlying MAMBAC is that if the data are categorical, there should be an optimal score that separates the two groups. In the current investigation, this would be an optimal number of cannabis dependence symptoms. J. Ruscio's MAMBAC program allows analyses with one variable serving as an output variable, while a composite of the remaining indicators serves as the input variable, thus providing more power than traditional MAMBAC procedures [23]. Our MAMBAC analysis therefore produced seven graphs, one for each indicator. We partitioned each composite input indicator into 50 evenly spaced cuts. At each cut, the variables are standardized and the difference score of the means on the output variable for those cases located above and below the cut are plotted on the y-axis. Taxonicity is indicated by a peak in the graph which represents the optimal point for maximally separating the two groups. Dimensional results appear concave and do not have a peak.

## Abbreviations

DSM *Diagnostic and Statistical Manual of Mental Disorders*

MAMBAC Mean Above Minus Below a Cut

MAXEIG Maximum Eigenvalue

NESARC National Epidemiologic Survey on Alcohol and Related Conditions

## Competing interests

The author(s) declare that they have no competing interests.

## Authors' contributions

TFD contributed to the study conceptualization, and writing, and performed the data analysis. ME contributed to the study conceptualization and writing. Both authors read and approved the final manuscript.
